# Recombinant human Thymosin β4 ameliorates experimental colitis and intestinal fibrosis through suppression of mineralocorticoid receptor signaling

**DOI:** 10.1186/s43556-026-00539-9

**Published:** 2026-08-17

**Authors:** Tao-ran Zhao, En-bo Hu, Meng-wei Wang, Yan-fang Zhai, Yun-yun Mao, Wen-yi Hou, Xiao-zheng Zhang, Zhi-zhen Liu, Shu-lin Hou, Jun-jie Xu, Rui Yu

**Affiliations:** 1 Department of Biochemistry and Molecular Biology, College of Basic Medical Sciences; Shanxi Key Laboratory of Birth Defect and Cell Regeneration; MOE Key Laboratory of Coal Environmental Pathogenicity and Prevention, Academy of Medical Sciences, Shanxi Medical University, Taiyuan, Shanxi China; 2 Shanxi Key Laboratory of Classical Prescription Yang-Strengthening, Taiyuan, Shanxi China; 3 National Key Laboratory of Advanced Biotechnology, Academy of Military Medical Sciences, Beijing, China; 4 School of Public Health, Shanxi Medical University, Taiyuan, Shanxi China

**Keywords:** Thymosin β4, Inflammatory bowel diseases, Experimental colitis, Intestinal fibrosis, Mineralocorticoid receptor, NR3C2

## Abstract

Inflammatory bowel disease (IBD) is a chronic inflammatory disorder characterized by epithelial barrier disruption, persistent mucosal inflammation, and progressive intestinal fibrosis, for which effective therapeutic options remain limited. Thymosin β4 (Tβ4) is a highly conserved endogenous peptide with established roles in tissue repair and immune regulation, but its contribution to IBD pathogenesis has not been fully elucidated. Here, we found that *TMSB4X*, the gene encoding Tβ4, was downregulated in colonic tissues of IBD patients. To investigate its functional significance, we generated *Tmsb4x*-deficient mice and demonstrated that loss of endogenous Tβ4 markedly increased susceptibility to dextran sulfate sodium (DSS)-induced colitis. Conversely, oral administration of recombinant human Tβ4 (rhTβ4) significantly improved survival, alleviated body weight loss, reduced epithelial injury, and suppressed inflammatory cytokine production in both prophylactic and therapeutic colitis models. Furthermore, rhTβ4 attenuated intestinal fibrosis, as evidenced by reduced expression of fibrosis-associated markers and decreased collagen I deposition. Transcriptomic analysis revealed that rhTβ4 partially restored DSS-induced gene dysregulation and suppressed mineralocorticoid receptor (MR, NR3C2) signaling, a pathway further supported by reporter assays and downstream target gene analyses. Collectively, these findings identify Tβ4 as an endogenous protective factor against intestinal inflammation and fibrosis and suggest that pharmacological restoration of Tβ4 activity may represent a promising therapeutic strategy for IBD through modulation of mineralocorticoid receptor signaling.

**Supplementary Information:**

The online version contains supplementary material available at 10.1186/s43556-026-00539-9.

## Introduction

Inflammatory bowel disease (IBD) is a chronic inflammatory disorder of the gastrointestinal tract, mainly comprising Crohn's disease (CD) and ulcerative colitis (UC) [1, 2]. Hallmark pathological features of IBD include dysregulated immune responses, disruption of the intestinal epithelial barrier, and chronic mucosal inflammation, all of which contribute to impaired intestinal homeostasis [2–4]. Sustained inflammation further promotes excessive extracellular matrix deposition and intestinal fibrosis, leading to luminal narrowing, stricture formation, and, ultimately, intestinal obstruction [5–7]. Importantly, currently available therapies primarily target inflammatory processes but have limited efficacy in preventing or reversing established fibrosis, leaving many patients dependent on endoscopic or surgical intervention [7–9]. Therefore, identification of endogenous protective mechanisms that simultaneously limit intestinal inflammation and fibrotic remodeling remains an important unmet need in IBD research.

The mineralocorticoid receptor (MR, encoded by NR3C2) is a ligand-activated nuclear receptor traditionally recognized for its role in electrolyte and fluid homeostasis. Aberrant MR signaling has been implicated in the development of fibrosis in multiple organs, including the heart [10, 11] and kidney [12, 13], and pharmacological inhibition of MR has shown therapeutic benefits in experimental fibrotic diseases [14, 15]. Recent studies have further implicated MR signaling in intestinal inflammation and fibrosis, identifying MR as a potential therapeutic target in IBD [11, 16, 17]. However, the endogenous mechanisms that restrain MR activation during intestinal injury remain poorly understood. Identification of physiological regulators capable of limiting aberrant MR signaling may therefore provide new therapeutic opportunities for controlling both intestinal inflammation and fibrotic remodeling.

Thymosin β4 (Tβ4) is an evolutionarily conserved peptide consists of 43 amino acid that is encoded by the *TMSB4X* gene and is widely expressed in mammalian tissues and body fluids [18, 19]. Beyond its established role in actin dynamics [20], Tβ4 has been demonstrated to possess diverse biological activities, including regulation of inflammation [21], inhibition of apoptosis and oxidative stress [22], promotion of tissue repair and enhancement of wound healing [23–25]. In the gastrointestinal tract, Tβ4 is produced by intestinal epithelial and immune cells and contributes to the maintenance of mucosal homeostasis [26–28]. Tβ4 has been reported to play a protective role in experimental colitis, as adenovirus-mediated overexpression of Tβ4 was shown to alleviate dextran sulfate sodium (DSS)- and 2,4,6-trinitrobenzenesulfonic acid (TNBS)-induced intestinal inflammation [28]. In addition, recent work has implicated Tβ4 in the regulation of autophagy and mucus barrier function, further supporting its involvement in intestinal biology [29]. However, these observations also indicate the context-dependent nature of Tβ4 signaling and highlight that its precise role in intestinal disease remains incompletely understood. Importantly, whether endogenous Tβ4 deficiency contributes to susceptibility to intestinal inflammation, whether Tβ4 regulates the development of intestinal fibrosis, and whether these effects involve modulation of MR signaling remain poorly characterized.

In the present study, we hypothesized that loss of endogenous Tβ4 would contribute to colitis susceptibility, whereas restoration of Tβ4 activity would alleviate intestinal inflammation and fibrosis through modulation of MR signaling. To test this hypothesis, we analyzed human IBD datasets, generated Tmsb4x-deficient mice, evaluated recombinant human Tβ4 (rhTβ4) in prophylactic and therapeutic DSS models, and performed transcriptomic and mechanistic analyses. This study identified Tβ4 as a previously underappreciated regulator of intestinal homeostasis and support its therapeutic potential in IBD.

## Results

### Reduced *TMSB4X* expression in colonic tissues from patients with IBD

**Fig. 1:**
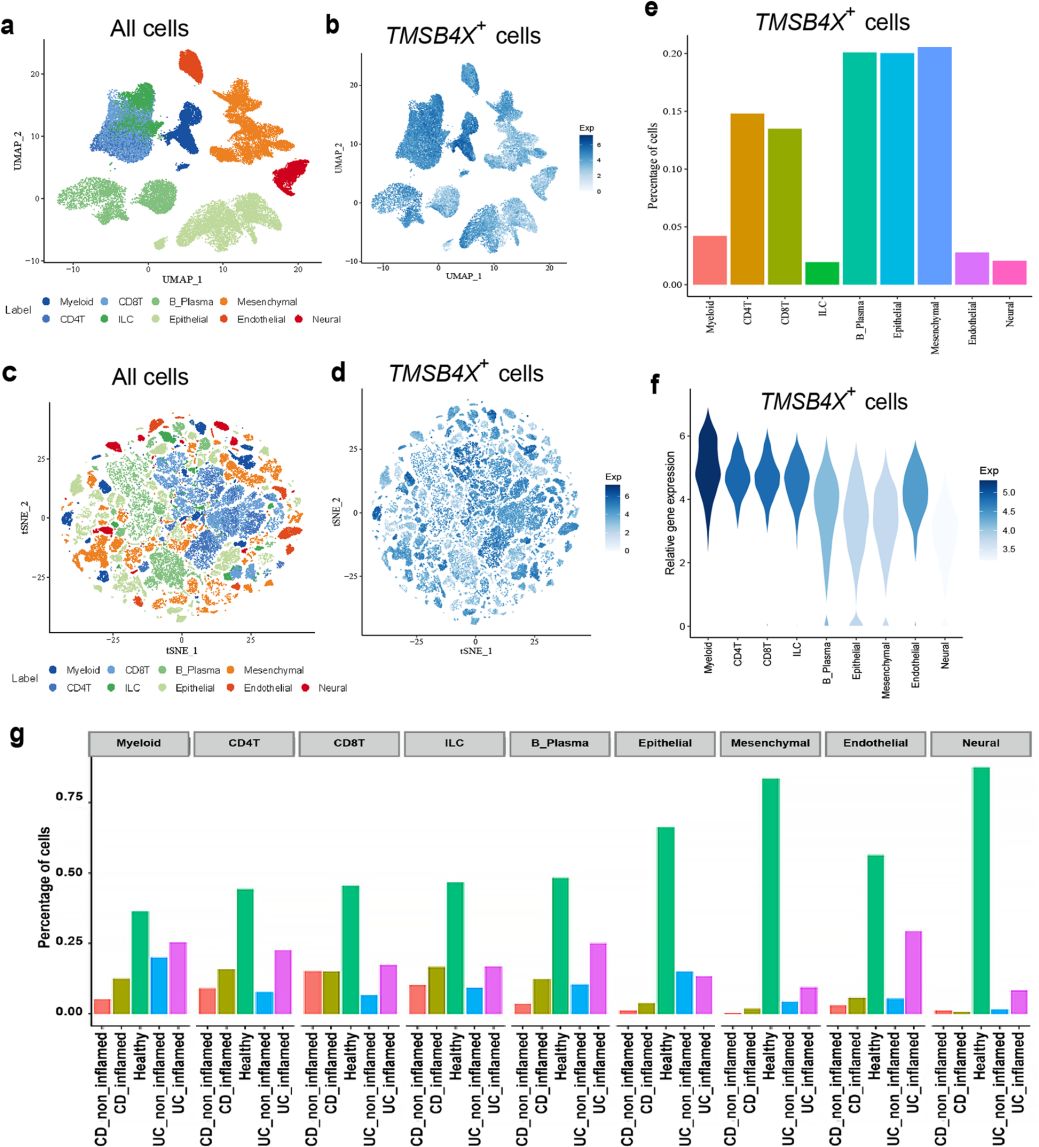
*TMSB4X* expression in colon tissues from healthy population and IBD patients. **a** UMAP projection of cells in colon tissue colored by cell type assignment. **b** Expression of *TMSB4X* across different cell types overlap with UMAP projection. **c** t-SNE projection of cells in colon tissue colored by cell type assignment. **d** Expression of *TMSB4X* across different cell types overlap with t-SNE projection. **e** Bar plot showing percentage of *TMSB4X* positive cells in different cell types. **f** Violin plot showing relative *TMSB4X* gene expression level in different cell types. **g** Bar plot showing percentage of *TMSB4X* positive cells in different cell types under healthy, CD or UC disease condition

To characterize the potential involvement of Tβ4 in human IBD, we initially analyzed the expression pattern of the Tβ4 encoding gene *TMSB4X*, using a publicly available single-cell RNA sequencing (RNA-seq) dataset comprising colonic tissues from healthy controls and patients with UC or CD. *TMSB4X* was broadly expressed across multiple cell populations in the human colon (Fig. 1a-d). Quantitative analysis of *TMSB4X*-positive cells revealed that mesenchymal cells, epithelial cells, plasma cells, CD4⁺ T cells, and CD8⁺ T cells contained the highest proportions of *TMSB4X*-positive cells (Fig. 1e), whereas myeloid cells, CD4⁺ and CD8⁺ T cells, innate lymphoid cells (ILCs), and endothelial cells exhibited the highest expression intensities (Fig. 1f).

We next compared *TMSB4X* expression between healthy individuals and IBD patients. As shown in Fig. 1g, *TMSB4X* expression displayed an overall decreasing trend in both inflamed and non-inflamed colonic tissues from patients with UC and CD across the major cellular compartments represented in the dataset. These findings suggest that *TMSB4X* is widely distributed in the human colon and its reduction is associated with IBD.

### Loss of Tβ4 increases susceptibility to DSS-induced experimental colitis model

Given the reduced expression of *TMSB4X* observed in colonic tissues from patients with IBD, we hypothesized that deficiency of endogenous Tβ4 would predispose to disease susceptibility and severity. To investigate the functional significance of Tβ4 deficiency in intestinal inflammation, we generated *Tmsb4x*-deficient (Tβ4^⁻/⁻^) mice. Prior to disease induction, as shown in Fig. S1, colon length and overall tissue architecture were comparable between Tβ4^−/−^ and wildtype (WT) mice (Fig. S1a, b). Assessment of epithelial proliferative activity using Ki67 immunostaining together with BrdU incorporation revealed no apparent differences between genotypes (Fig. S1c). In addition, expression of the goblet cell marker Muc2 was preserved in Tβ4^*−/−*^ mice (Fig. S1c). Consistent with these findings, colonic levels of the pro-inflammatory cytokines IFN-γ, TNF, IL-6, and IL-1β were not significantly different from those observed in WT mice (Fig. S1d). Collectively, these results indicate that constitutive loss of Tβ4 does not result in overt disruption of intestinal homeostasis or spontaneous intestinal inflammation under basal conditions.

**Fig. 2:**
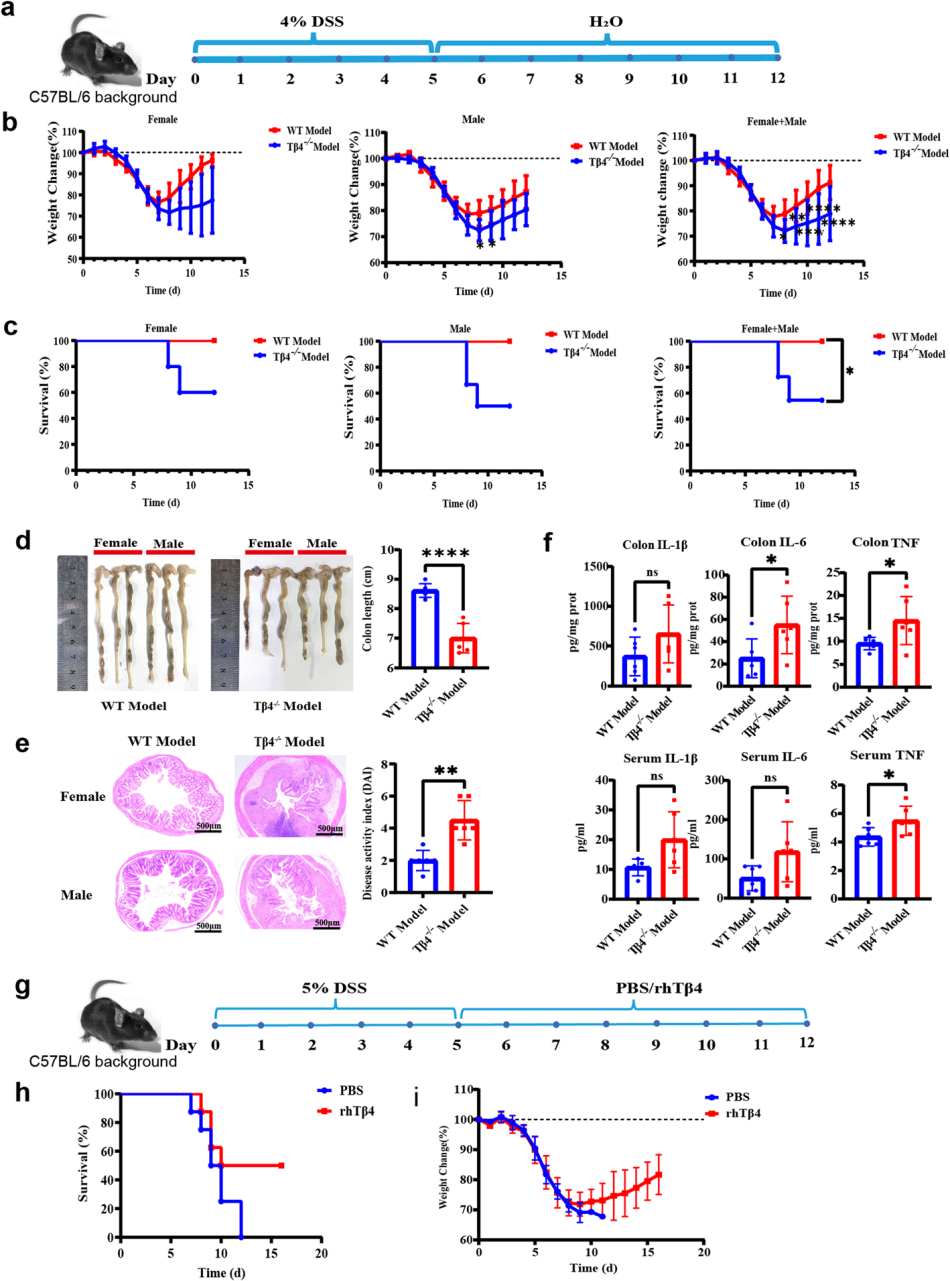
Increased susceptibility of Tβ4-deficient mice to colitis. **a** Schematic of acute colitis induction in WT and Tβ4^⁻/⁻^ mice by administration of 4% DSS in drinking water for 5 days followed by normal water. **b** Body weight changes after DSS exposure (*n* = 5 females and 6 males per group). **c** Survival curves of WT and Tβ4^⁻/⁻^ mice after DSS treatment (*n* = 5 females and 6 males per group). **d** Representative images and quantification of colon length on day 12 (*n* = 3 females and 3 males per group). **e** Representative H&E staining of colon sections and disease activity index (DAI) scores on day 12 (*n* = 3 females and 3 males per group). **f** Levels of IL-1β, IL-6, and TNF measured in serum and colon tissues (*n* = 3 females and 3 males per group). **g** Experimental design for DSS-induced colitis and therapeutic administration of rhTβ4 in Tβ4^⁻/⁻^ mice. Mice received 5% DSS in drinking water for 5 days, followed by normal water for 7 days. rhTβ4 or PBS was administered daily by oral gavage (*n* = 8 males per group). **h** Survival of WT and Tβ4^⁻/⁻^ mice after DSS with or without rhTβ4 treatment. **i** Body weight changes in WT and Tβ4^⁻/⁻^ mice after DSS with or without rhTβ4 treatment. Data are presented as mean ± SD. *p < 0.05, **p < 0.01, and ***p < 0.001; ns, no significance

To determine the contribution of endogenous Tβ4 in colitis, WT and Tβ4^⁻/⁻^ mice were administered 4% DSS for 5 days (Fig. 2a). Compared with WT controls, Tβ4^⁻/⁻^ mice exhibited greater body weight loss and slower recovery (Fig. 2b), as well as higher mortality rates (Fig. 2c), with no significant sex-dependent differences observed.

Macroscopic examination on day 12 revealed marked colon shortening in both male and female Tβ4^⁻/⁻^ mice (Fig. 2d). Histological analysis further demonstrated more severe epithelial destruction, increased intestinal wall edema, and pronounced luminal narrowing compared with WT mice (Fig. 2e). Additionally, Tβ4^⁻/⁻^ mice exhibited elevation of inflammatory mediators, including IL-1β, IL-6, and TNF, in both colonic tissues and serum (Fig. 2f).

To determine whether restoration of Tβ4 activity could rescue this phenotype, Tβ4^⁻/⁻^ mice were treated with rhTβ4 following DSS exposure. rhTβ4 supplementation significantly improved survival (Fig. 2g, h) and promoted recovery of body weight (Fig. 2i) compared with untreated Tβ4^⁻/⁻^ mice.

Taken together, these findings demonstrate that endogenous Tβ4 protects against DSS-induced colitis, whereas loss of Tβ4 markedly increases susceptibility to intestinal inflammation and tissue injury.

## rhTβ4 treatment alleviates experimental colitis

To determine whether exogenous Tβ4 could prevent or treat colitis, rhTβ4, which shares a highly conserved amino acid sequence with murine Tβ4, was evaluated in both prophylactic and therapeutic DSS-induced colitis models. Because peptide stability and bioavailability are important considerations for oral administration, we first compared the biodistribution of Cy5.5-labeled rhTβ4 following oral gavage and intraperitoneal injection. Oral administration resulted in prolonged intestinal retention and higher local fluorescence intensity, whereas systemic distribution was limited compared with intraperitoneal delivery (Fig. S2). In vivo fluorescence imaging demonstrated distinct biodistribution patterns following oral gavage and intraperitoneal administration of rhTβ4-Cy5.5. After oral administration, fluorescence signals were predominantly localized to the stomach and intestinal tract throughout the observation period (Fig. S2a, b). In contrast, intraperitoneal injection resulted in a broader systemic distribution, with detectable fluorescence signals in multiple organs and tissues (Fig. S2d, e). Quantitative analysis of intestinal fluorescence intensity revealed that orally administered rhTβ4 rapidly accumulated in the gastrointestinal tract and reached a peak signal of approximately 1.5 × 10^12^ [p/s]/[μW/cm^2^] at 1 h post-administration (Fig. S2c). Notably, intestinal fluorescence remained above 5 × 10^11^ [p/s]/[μW/cm^2^] for more than 9 h, indicating prolonged local retention of rhTβ4 within the intestine. By comparison, intraperitoneal administration produced a substantially lower intestinal fluorescence peak of approximately 5 × 10^11^ [p/s]/[μW/cm^2^], which was maintained for only about 3 h before declining below 4 × 10^11^ [p/s]/[μW/cm^2^] (Fig. S2f). These findings demonstrate that oral administration provides higher and more sustained intestinal exposure to rhTβ4 than intraperitoneal injection, supporting its use in subsequent studies of experimental colitis.

**Fig. 3:**
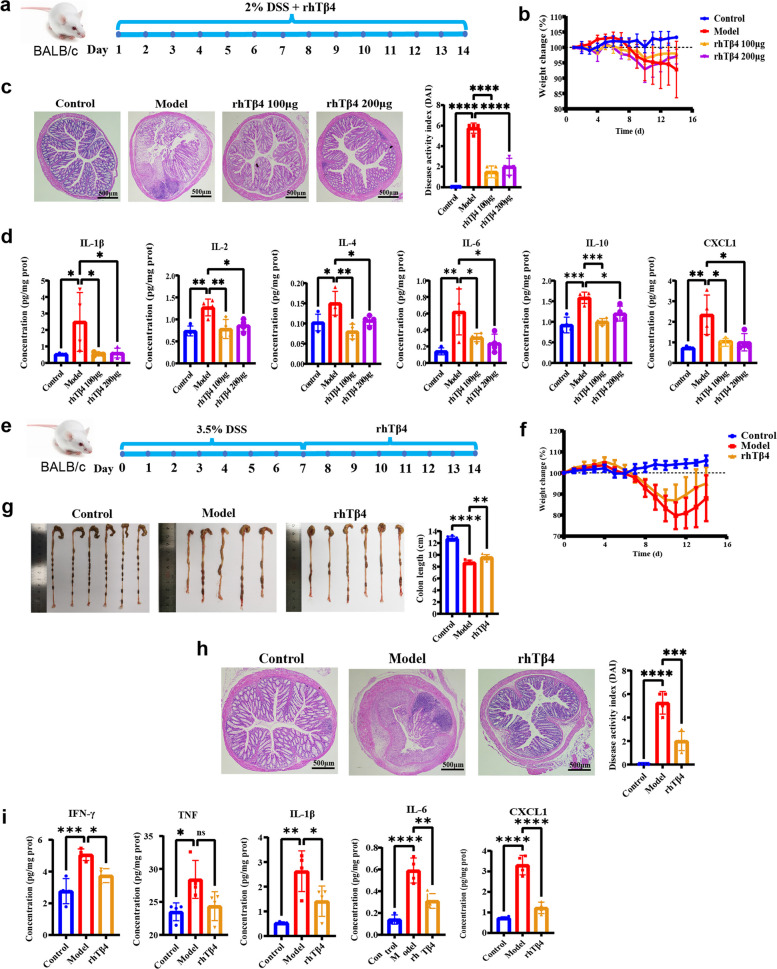
rhTβ4 ameliorates colonic injury in DSS-induced colitis. **a** Experimental design of prophylactic treatment. Mice received 2% DSS in drinking water for 14 days and were gavaged daily with PBS, rhTβ4 (100 μg), or rhTβ4 (200 μg) (*n* = 4 per group). **b** Body weight changes during 2% DSS administration. **c** Representative H&E staining of colon sections and disease activity index (DAI) scores on day 14 (*n* = 4 per group). **d** Cytokine levels (IL-1β, IL-2, IL-4, IL-6, IL-10, CXCL1) in colon tissues on day 14 (*n* = 4 per group). **e** Experimental design of therapeutic treatment. Mice received 3.5% DSS in drinking water for 7 days followed by normal water. Groups were: Control (no DSS, PBS gavage), Model (DSS + PBS gavage), and rhTβ4 (DSS + 100 μg rhTβ4 gavage daily) (*n* = 6 per group). **f** Body weight changes after 3.5% DSS administration. **g** Representative images and quantification of colon length on day 14 (*n* = 6 per group; Model group, *n* = 5 due to one death). **h** Representative H&E staining of colon sections and DAI scores on day 14 (*n* = 4 per group). **i** Cytokine levels (IFN-γ, TNF, IL-1β, IL-6, CXCL1) in colon tissues on day 14 (*n* = 4 per group). Data are presented as mean ± SD. *p < 0.05, **p < 0.01, and ***p < 0.001; ns, no significance

In the prophylactic model, mice received 2% DSS together with daily oral administration of rhTβ4 (100 or 200 μg; Fig. 3a). Both doses attenuated DSS-induced body weight loss, with the 100 μg dose showing superior efficacy (Fig. 3b). Histological examination on day 14 demonstrated that DSS caused crypt loss, epithelial injury, and submucosal edema, whereas rhTβ4 treatment preserved epithelial architecture and reduced tissue edema (Fig. 3c). Notably, submucosal edema was less severe in the 100 μg rhTβ4 treatment group compared with the 200 μg group (Fig. 3c). Consistent with these findings, rhTβ4 significantly decreased the colonic levels of multiple pro-inflammatory cytokines, including IL-1β, IL-2, IL-6, and the neutrophil chemoattractant CXCL1 (Fig. 3d). Together, these results indicate that prophylactic rhTβ4 administration protects against DSS-induced intestinal injury and inflammation.

We next evaluated the therapeutic efficacy of rhTβ4 in mice with established colitis induced by 3.5% DSS (Fig. 3e). Oral administration of 100 μg of rhTβ4 significantly promoted weight recovery (Fig. 3f), reduced intestinal bleeding, and restored colon length (Fig. 3g). Histological analysis revealed restoration of epithelial integrity, preservation of crypt structure, and reduced intestinal edema following rhTβ4 treatment (Fig. 3h). In parallel, rhTβ4 markedly inhibited the production of those pro-inflammatory cytokines (Fig. 3i). Taken together, these findings suggest that rhTβ4 provides both preventive and therapeutic benefits in experimental colitis.

## rhTβ4 treatment attenuates colonic transcriptomic alterations in experimental colitis

To elucidate the molecular mechanisms responsible for therapeutic action of rhTβ4, we performed RNA-seq on colonic tissues collected from control, DSS-treated, and rhTβ4-treated mice in the therapeutic IBD model.

**Fig. 4:**
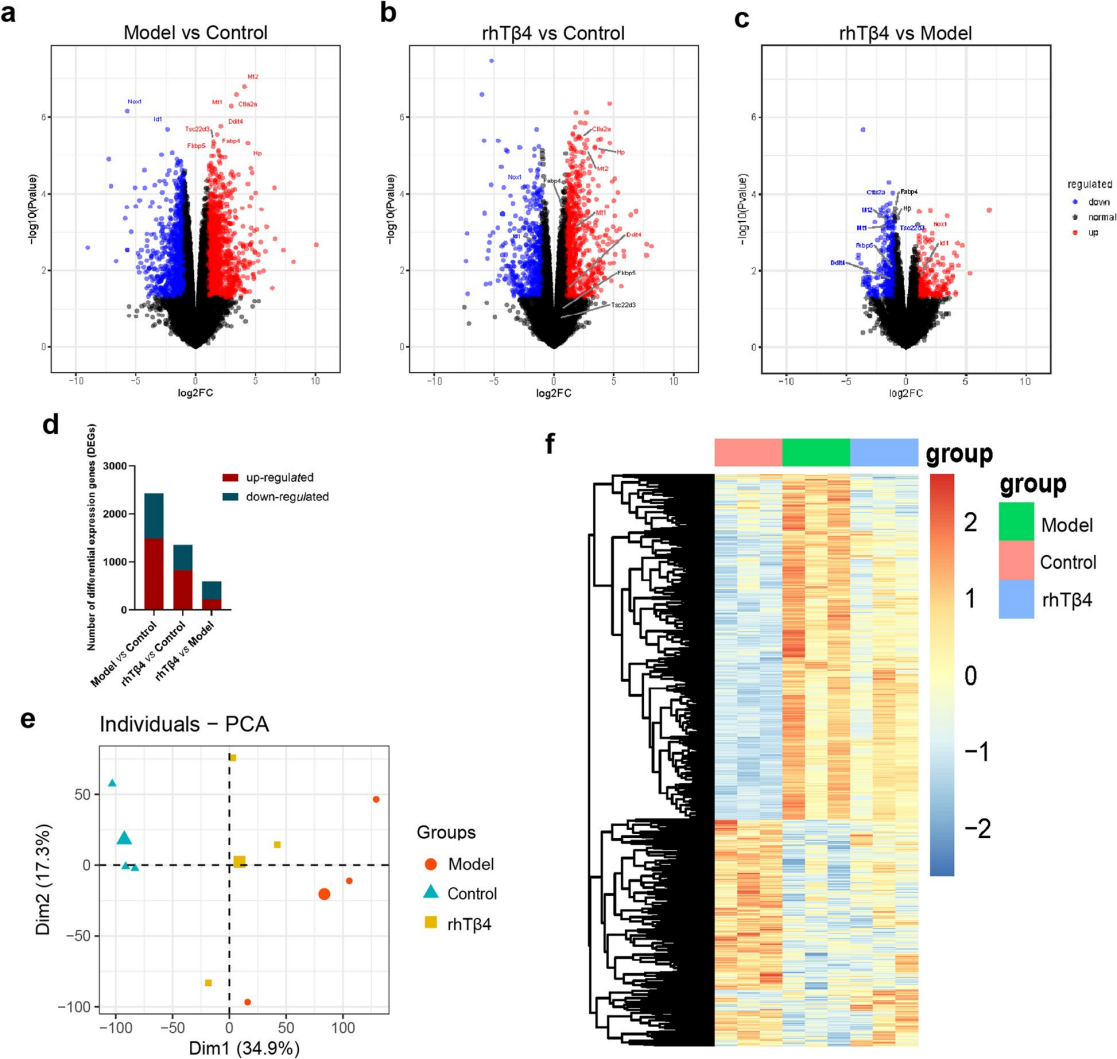
rhTβ4 treatment reduced colon tissue gene expression profiles change in DSS induced colitis. **a**, **b** and **c** Volcano plots showing the distribution of differentially expressed genes (DEGs). Red dots indicate upregulated genes, while blue dots represent downregulated genes (*p*-adjust < 0.05, |log₂ fold change|> 1). **a** DEGs in the model group compared to the control group. **b** DEGs in the rhTβ4 treated group compared to the control group. **c** DEGs in the rhTβ4 treated group compared to the model group. **d** Bar chart summarizing the number of upregulated and downregulated DEGs across comparisons. **e** Principal component analysis (PCA) showing the clustering of mRNA expression profiles in colon tissues. **f** Heatmap showing hierarchical clustering of DEGs across samples, highlighting distinct transcriptional patterns between the model and rhTβ4 treated groups

Differential expression analysis identified substantial transcriptomic alterations following DSS administration. Compared with control mice, DSS treatment induced 2,431 differentially expressed genes (DEGs), comprising 1,491 genes upregulated and 940 genes downregulated (Fig. 4a, d). In contrast, rhTβ4-treated mice exhibited fewer dysregulated genes, with 832 upregulated and 521 downregulated DEGs relative to controls mice (Fig. 4b, d). Direct comparison between DSS-treated and rhTβ4-treated groups identified 218 upregulated and 377 downregulated genes following rhTβ4 treatment (Fig. 4c, d). Notably, the ten most significantly dysregulated genes in DSS-treated mice showed partial or complete normalization after rhTβ4 administration (Fig. 4a–c). Principal component analysis (PCA) plot showed distinct separation between DSS-treated and control mice, whereas the rhTβ4-treated group shifted toward the control cluster (Fig. 4e). Consistently, hierarchical clustering analysis showed that rhTβ4 substantially restored DSS-induced alterations in gene expression across the transcriptome (Fig. 4f).

Overall, these results indicate that rhTβ4 partially reverses the widespread transcriptional dysregulation induced by DSS, supporting its role in restoring intestinal homeostasis at the molecular level.

## rhTβ4 suppresses fibrosis-associated transcriptional programs in experimental colitis

To explore the signaling pathways associated with the protective effects of rhTβ4, we performed Gene Ontology (GO) enrichment analysis using DEGs identified by RNA-seq.

**Fig. 5:**
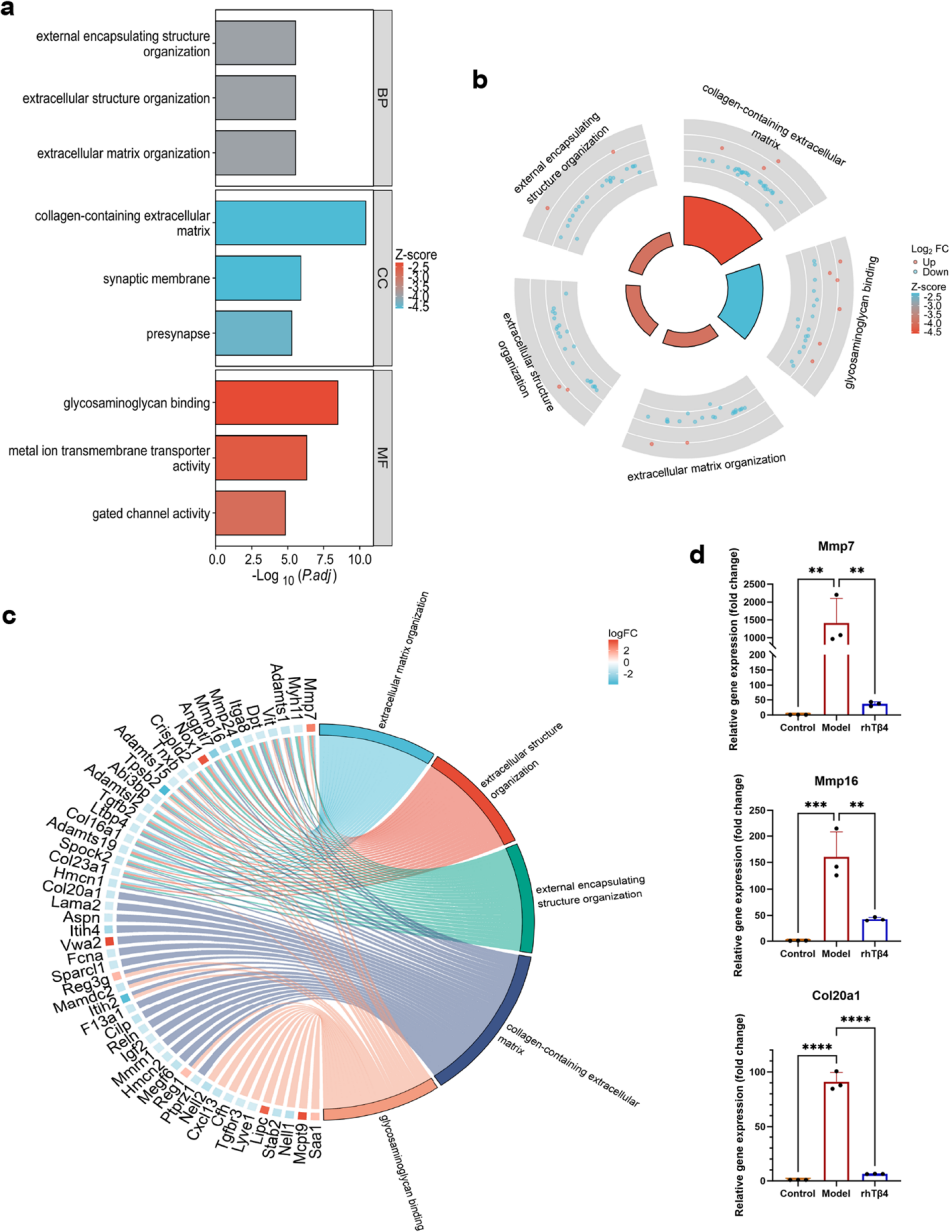
rhTβ4 Treatment suppressed intestinal fibrosis induced by DSS. **a** The most significantly enriched biological processes (BP), molecular function (MF) and cellular component (CC) emerging from the DEGs between rhTβ4 treated and model groups are graphed according to the − log_10_ of the *p*-value. The color of each bar indicates the stand score (z-score). **b** The tissue fibrosis related pathways among the most significantly enriched Gene Ontology (GO) terms. The color of the inner trapezoid indicates the *p*-value of each GO term. The color and distance to the inner edge of the dots in the outer trapezoid indicate the regulated form and fold change of the involved DEGs, respectively, of each GO term. **c** The chord diagram showing relationship between the tissue fibrosis related pathways among the most significantly enriched GO terms and the DEGs enriched in these pathways. Lines of different colors represent different pathway names, with gene names on the left side. **d** Relative mRNA levels of *Mmp7, Mmp9 and Col20a* in colon tissue detected by qPCR (*n* = 3). Data are presented as mean ± SD. *p < 0.05, **p < 0.01, and ***p < 0.001; ****p < 0.0001; ns, no significance

The top enriched terms from the biological process, cellular component, and molecular function categories are shown in Fig. 5a. Notably, several significantly enriched pathways were associated with extracellular matrix (ECM) remodeling and fibrosis, including collagen-containing extracellular matrix, extracellular matrix organization, extracellular structure organization, and glycosaminoglycan binding. Because intestinal fibrosis is a major complication of IBD, these findings suggest that rhTβ4 may influence fibrotic remodeling.

Further analysis demonstrated that rhTβ4 treatment largely reversed DSS-induced upregulation of genes within these fibrosis-related pathways (Fig. 5b). Chord mapping further identified several representative genes involved in these pathways, including *Mmp7*, *Mmp16*, *Myh11*, *Col20a1*, and *Reg3g*, highlighting potential downstream effectors mediating the anti-fibrotic effects of rhTβ4 (Fig. 5c).

To validate the transcriptomic findings, quantitative PCR (qPCR) was performed on selected candidate genes. Consistent with the RNA-seq results, rhTβ4 significantly attenuated the DSS-induced upregulation of *Mmp7*, *Mmp16*, *Col20a1*, and restored *Reg3g* expression (Fig. 5d).

Taken together, these data indicate that rhTβ4 suppresses fibrosis-associated transcriptional programs in DSS-induced colitis, supporting a potential role in limiting intestinal fibrotic remodeling.

## rhTβ4 attenuates intestinal fibrosis in experimental colitis

**Fig. 6:**
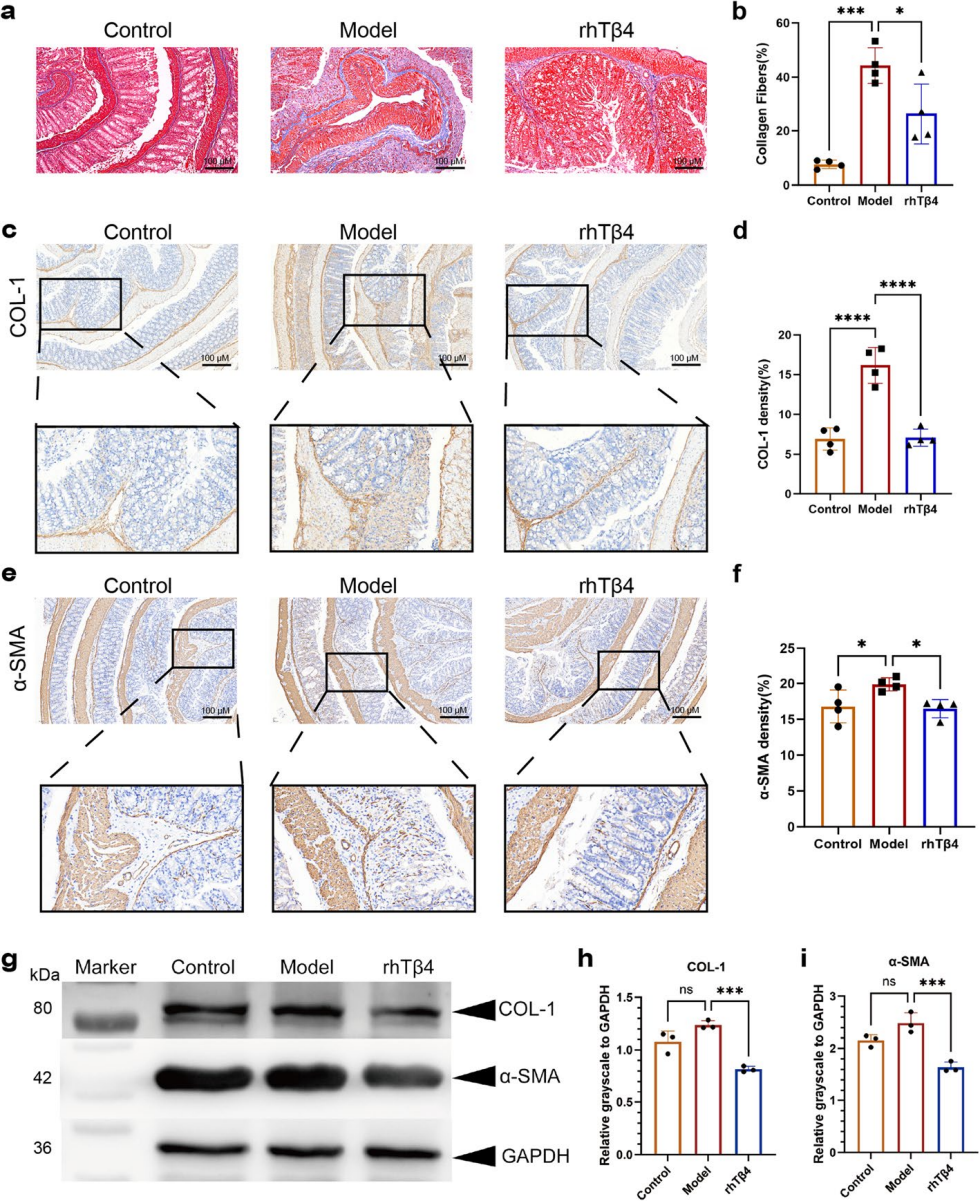
rhTβ4 treatment inhibits the high levels of fibrosis markers in histological assays. **a** Representative image of the Masson trichrome-stained colon sections: collagen fibers deposition (blue area). **b** Quantitative analysis of the collagen fibers -positive area (*n* = 4). **c** Representative immunohistochemical staining for collagen I in colon sections. **d** Quantitative analysis of the collagen I- positive area (*n* = 4). **e** Representative immunohistochemical staining for α-SMA in colon sections. **f** Quantitative analysis of the α-SMA—positive area (*n* = 4). **g** to **i** Western blot analysis of collagen I and α-SMA protein expression in colons. Representative western blotting results (**g**) and relative grayscale of collagen I (**h**) and α-SMA (**i**) (*n* = 3). Data are presented as mean ± SD. *p < 0.05, **p < 0.01, and ***p < 0.001; ns, no significance

To determine whether rhTβ4 attenuates intestinal fibrosis, colonic tissues from DSS-induced colitis model mice were subjected to histological and molecular analyses. Masson’s trichrome staining revealed extensive ECM accumulation in DSS-treated mice relative to controls. In contrast, rhTβ4 treatment markedly reduced collagen-positive fibrotic areas in the colon (Fig. 6a, b).

Consistently, immunohistochemical analysis revealed significantly lower protein levels of collagen I and α-SMA in the rhTβ4-treated group compared with the model group (Fig. 6c–f). These findings were further validated by western blot analysis. Consistent with the immunohistochemical findings, rhTβ4 markedly decreased the amount of collagen I and α-SMA compared with the DSS-treated group (Fig. 6g–i).

Taken together, histological and biochemical analyses consistently demonstrate that rhTβ4 attenuates intestinal fibrotic remodeling in DSS-induced colitis.

## rhTβ4 suppresses mineralocorticoid receptor signaling and downstream profibrotic responses

**Fig. 7:**
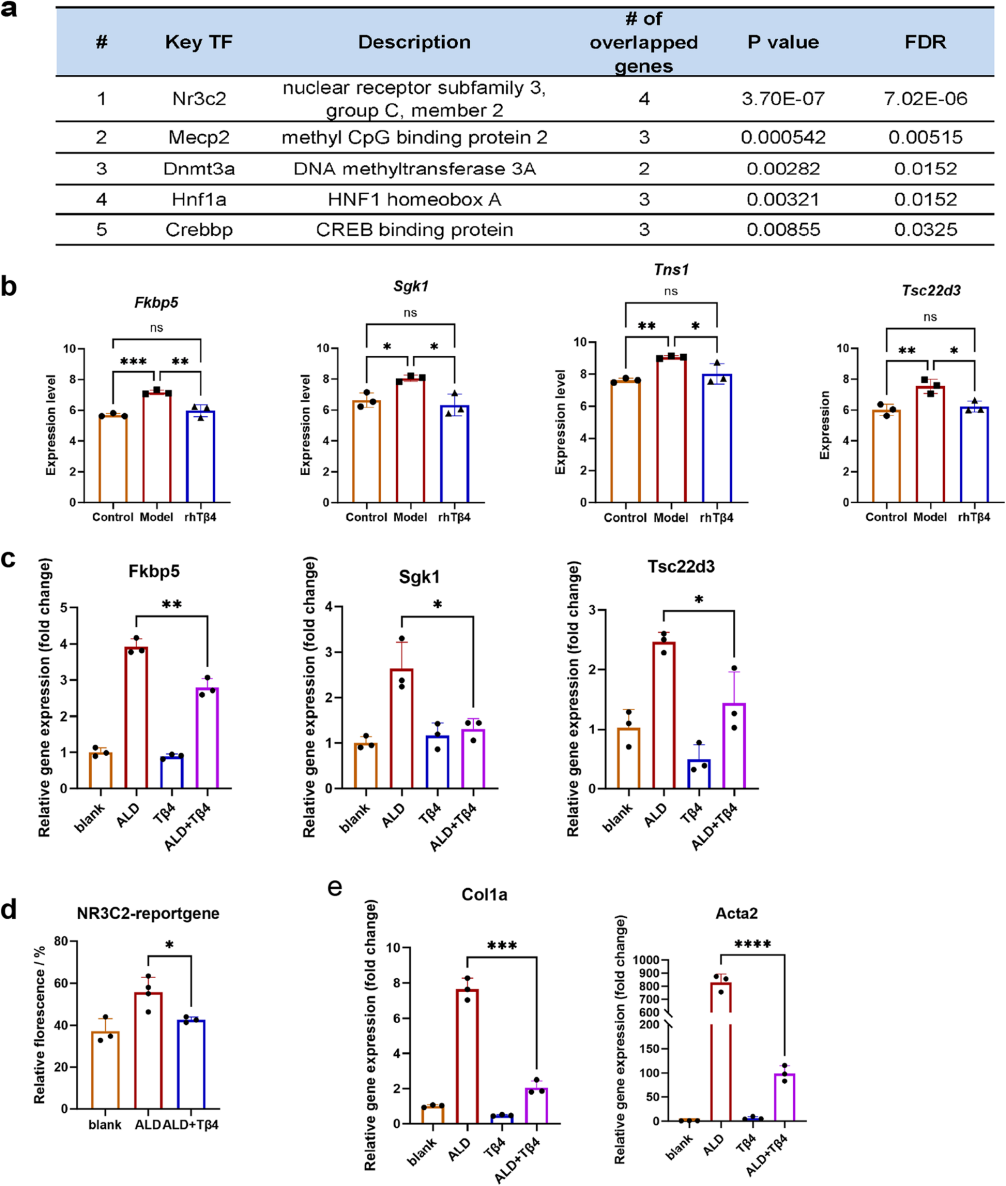
rhTβ4 suppresses mineralocorticoid receptor signaling and downstream profibrotic responses. **a** Top five transcription factors identified by TRRUST analysis based on DEGs between rhTβ4-treated or colitis mouse models. **b** Violin plots showing the expression levels of NR3C2 target genes in colon tissues from Control, Model, and rhTβ4 groups. **c** Relative mRNA levels of *Fkbps*, *Sgk1* and *Tsc22d3* in MCFCs treated with aldosterone (ALD), rhTβ4 alone or their combination for 24 h (*n* = 3). **d** Relative florescence of NR3C2-reportgene of in MCFCs treated with ALD alone or in combination with rhTβ4 for 24 h (*n* = 3). **e** Relative mRNA levels of *Col1a* and *Acta2* in MCFCs treated with ALD, rhTβ4 alone or their combination for 24 h (*n* = 3). Data are presented as mean ± SD. *p < 0.05, **p < 0.01, and ***p < 0.001; ****p < 0.0001; ns, no significance

To identify key transcriptional regulators associated with the protective effects of rhTβ4, we performed transcription factor enrichment analysis on DEGs between DSS-treated and rhTβ4 -treated groups using the TRRUST database. Among the predicted regulators, MR (encoded by *Nr3c2*), a ligand-activated nuclear receptor implicated in inflammation and fibrosis, emerged as the most significantly enriched transcription factor (Fig. 7a).

Consistent with this finding, DSS treatment markedly increased the expression of several established MR target genes, including *Fkbp5*, *Sgk1*, *Tns2*, and *Tsc22d3* [30], whereas rhTβ4 administration largely restored their expression toward basal levels (Fig. 7b). To substantiate the relevance of MR activation in human UC, the expression patterns of its downstream target genes were analyzed using publicly accessible transcriptomic profiles derived from intestinal specimens of patients with UC, CD, and healthy controls (Fig. S3). In dataset GSE87466, four MR-responsive genes—*SGK1, RASL12, FKBP5,* and *TSC22D3*—were detectable (Fig. S3a). Except for *SGK1*, the other three genes exhibited a clear upregulation trend in UC samples relative to healthy controls.

Analysis of dataset GSE75214, which includes both colonic and ileum samples from individuals with UC and CD, revealed detectable expression of all five MR target genes (Fig. S3b). Interestingly, with the exception of *SGK1*, the other four genes exhibited a consistent upregulation in expression specifically in colonic tissues from IBD patients, whereas no significant alteration were identified in the ileum samples. These findings suggest a colon-predominant activation pattern of MR-associated transcriptional programs in human IBD.

To directly evaluate the effect of rhTβ4 on MR signaling, mouse colon fibroblasts (MCFCs) were stimulated with aldosterone, a canonical MR agonist. Aldosterone significantly induced the upregulation of the MR target genes *Fkbp5*, *Sgk1*, and *Tns2*, while co-treatment with rhTβ4 markedly attenuated these responses (Fig. 7c). Consistently, dual-luciferase reporter assays demonstrated that aldosterone enhanced MR transcriptional activity, whereas rhTβ4 significantly suppressed this activation (Fig. 7d).

Because MR signaling has been implicated in fibrotic remodeling, we further assessed fibrosis-associated markers in MCFCs. Aldosterone stimulation increased the abundance of *Col1a* and *Acta2* (the gene encoding α-SMA). Notably, rhTβ4 effectively reversed the induction of both fibrotic markers (Fig. 7e).

Collectively, these findings demonstrate that rhTβ4 suppresses MR transcriptional activity, inhibits downstream target gene expression, and attenuates profibrotic responses, supporting MR signaling as a key mechanism underlying the protective effects of rhTβ4 in experimental colitis and intestinal fibrosis.

## Discussion

Tβ4 is a highly conserved peptide involved in tissue repair and immune regulation. Here, we demonstrate that *TMSB4X* expression is reduced in colonic tissues from patients with IBD and that Tmsb4x deficiency increases susceptibility to DSS-induced colitis, indicating a protective role for endogenous Tβ4 in intestinal homeostasis. Conversely, administration of recombinant human Tβ4 significantly improved survival, reduced intestinal injury, and suppressed inflammatory responses in experimental colitis. Transcriptomic analyses further showed that rhTβ4 partially restored DSS-induced transcriptional alterations. Together, these findings suggest Tβ4 as a protective regulator of intestinal inflammation and support the therapeutic potential of rhTβ4 in IBD.

Current mainstream therapies for IBD, including aminosalicylates, corticosteroids, and biologics, primarily target inflammatory pathways, yet have limited efficacy in preventing or reversing intestinal fibrosis, a major driver of stricture formation, obstruction, and surgical interventions in IBD patients [2, 3]. Fibrosis develops in parallel with chronic inflammation through ECM deposition during tissue repair [31, 32]. Although anti-inflammatory treatment can suppress inflammatory activity, fibrotic pathways may remain activated even after inflammation subsides, indicating that fibrosis can progress through partially inflammation-independent mechanisms [33]. Therefore, therapies that directly target intestinal fibrosis remodeling remains an important unmet clinical need in IBD.

In support of this unmet therapeutic need, our findings indicate that rhTβ4 possesses significant anti-fibrotic activity in experimental colitis. Transcriptomic analysis revealed that rhTβ4 reversed fibrosis-associated gene expression changes induced by DSS, with enrichment analyses highlighting suppression of ECM organization pathways. Consistent with these observations, histological and protein analyses demonstrated reduced collagen I and α-SMA expression following rhTβ4 treatment, supporting attenuation of intestinal fibrogenic responses. These findings extend the established anti-inflammatory and tissue-reparative functions of Tβ4 and suggest a previously underappreciated role in limiting intestinal fibrosis [23, 24, 34, 35]. Moreover, the favorable safety profile reported in preclinical and early clinical studies [36, 37], together with the feasibility of large-scale peptide production [38], further support the clinical potential of rhTβ4 as a therapeutic approach for IBD-associated fibrosis.

MR (NR3C2) is increasingly recognized as a central regulator linking inflammation signaling to tissue fibrotic remodeling. While classically studied in renal electrolyte homeostasis [39], aberrant MR activation has recently been implicated in experimental colitis and intestinal fibrogenesis [40]. In this study, multiple evidences support NR3C2 as a downstream target of Tβ4. rhTβ4 suppressed the expression of canonical NR3C2-responsive genes, inhibited NR3C2 reporter activity, and attenuated aldosterone-induced profibrotic responses in colonic fibroblasts. These findings suggest that rhTβ4 not only dampens intestinal inflammation but also interferes with MR-driven fibrotic signaling. To our knowledge, this is the first study linking Tβ4 to the regulation of MR activity in the context of intestinal inflammation and fibrosis.

Interestingly, rhTβ4 exhibited a nonlinear dose–response relationship in the prophylactic model, with the 100 μg dose producing greater protection than the 200 μg dose. Although the underlying mechanism remains unclear, this finding is consistent with the biphasic or hermetic-like responses reported for several endogenous regulatory peptides and may reflect context-dependent signaling or compensatory feedback mechanisms [41]. In addition, rhTβ4 reduced not only pro-inflammatory cytokines but also IL-4 and IL-10 levels. Given that IL-4 and IL-10 are frequently elevated as a compensatory response during active intestinal inflammation [42, 43], their reduction following rhTβ4 treatment may reflect normalization of the inflammatory milieu rather than nonspecific immunosuppression. Together, these observations suggest that Tβ4 functions as an immunomodulatory regulator rather than a simple inhibitor of immune responses.

These findings should nevertheless be interpreted in light of several important considerations. First, the human data were derived primarily from public transcriptomic datasets, and direct validation in clinical samples was not performed. Second, the constitutive *Tmsb4x* knockout model used in this study does not allow determination of the relative contributions of specific cellular compartments, such as epithelial, immune, or mesenchymal cells. Third, though DSS-induced colitis mimics key features of intestinal inflammation and post-inflammatory fibrosis, it does not completely reproduce the chronic fibrostenotic pathology observed in human IBD; therefore, the anti-fibrotic effects of rhTβ4 should be interpreted with caution. Finally, while our data support suppression of NR3C2 signaling as a key mechanism underlying the protective effects of rhTβ4, additional studies are required to define the upstream regulatory mechanisms and its interaction with established profibrotic pathways, including TGF-β signaling.

## Conclusions

In summary, Tβ4 functions as an endogenous protective factor in intestinal inflammation and fibrosis. rhTβ4 ameliorated experimental colitis, attenuated fibrotic remodeling, and suppressed NR3C2 signaling. These findings provide mechanistic insight into the role of Tβ4 in IBD and support the Tβ4–NR3C2 axis as a potential therapeutic target for intestinal inflammation and fibrosis.

## Materials and methods

### Reagents and antibodies

DSS, (molecular weight 36–50 kDa, Cat#160,110) was obtained from MP Biomedicals, CA, USA; 5-bromo-2′-deoxyuridine (BrdU, 40204ES80) were obtained from Yeasen Biotechnology, Shanghai, China. rhTβ4 was developed and produced in our laboratory [38].

Antibodies including anti-Ki-67 (GB121141), anti-MUC2 (GB11344), anti-BrdU (GB12051), anti-collagenⅠ (GB11022) and anti-α-SMA (GB111364) were all purchased from Servicebio, Wuhan, China.

### Single-Cell Analysis

Single-cell RNA sequencing (scRNA-seq) data used in this study were obtained from previously published datasets integrated in the scIBD (http://scibd.cn/, accessed on 23 May 2025) resource, which is a public database developed to collecting, analyzing, and visualizing scRNA-seq data from human IBD [44]. The datasets used in present study included samples from adult patients with ulcerative colitis (UC), Crohn’s disease (CD), and healthy controls. Specifically, the analysis incorporated the following publicly available datasets: GSE116222, GSE114374, GSE148837, SCP259, GSE134809, GSE157477, GSE125527, and SDY1765, derived from independent studies. See Table S1 for details. These datasets comprised intestinal tissue samples including colon, ileum, cecum, and rectum obtained from both inflamed and non-inflamed regions of IBD patients, as well as healthy controls. In total, 213 samples were included across all datasets. All patient-related clinical information, including disease classification and sample origin, was based on the annotations provided in the original publications. Downstream analyses were performed using the processed data as curated in the scIBD platform.

### Animal husbandry and ethics statement

All mice were maintained under specific pathogen-free (SPF) conditions in individually ventilated cages with controlled temperature (22 ± 2 °C), humidity (55 ± 10%), and a 12 h light/dark cycle, with ad libitum access to standard chow and water. All experimental procedures were approved by the Animal Laboratory of Laboratory Animal Center, Academy of Military Medical Sciences, and conducted in accordance with the National Institutes of Health Guide for the Care and Use of Laboratory Animals. Sample sizes were determined based on preliminary experiments, previous experience with DSS-induced colitis models, and published studies employing similar endpoints.

### Generation and genotyping of *Tmsb4x*-deficient mice

*Tmsb4x*-deficient (Tβ4^⁻/⁻^) mice were generated by co-injecting single-guide RNAs (sgRNAs) targeting the *Tmsb4x* gene together with Cas9 mRNA into fertilized mouse zygotes. Founder F0 mice were screened by PCR amplification of the target locus followed by Sanger sequencing. Positive F0 animals were crossed with wild-type (WT) mice to generate F1 offspring, whose genotypes were confirmed by PCR analysis. Heterozygous F1 mice were intercrossed to obtain homozygous Tβ4^⁻/⁻^ mice.

For routine genotyping, genomic DNA was extracted from tail biopsies and subjected to PCR analysis to distinguish WT, heterozygous, and homozygous knockout mice. PCR products were separated by agarose gel electrophoresis and interpreted according to the expected banding patterns. Using genotyping primer set 1, a 467-bp PCR product was specifically amplified from the Tmsb4x knockout allele, whereas no amplification product was detected in WT mice. In contrast, genotyping primer set 2 generated a 617-bp PCR product from the WT allele but did not produce a detectable band in homozygous knockout mice. Heterozygous animals exhibited both PCR products. Representative genotyping results and the corresponding interpretation criteria are shown in Fig. S4. The primer sequences were as follows:

- Genotyping primer set 1: forward primer, TGGAAATGGCTTCGATCTATCCAG; reverse primer, TTCAGCCTTGCTGAGATATTGTGT; expected product size, 467 bp.
- Genotyping primer set 2: forward frimer, GGAGTTTTGGAAAGCCAACATTGA; reverse primer, TTCAGCCTTGCTGAGATATTGTGT; expected product size, 617 bp.

### Establishment of experimental colitis model and rhTβ4 treatment in Tβ4^⁻/⁻^mice

To evaluate whether supplementation with Tβ4 could rescue the colitis phenotype in Tβ4^⁻/⁻^ mice, 8-week-old male Tβ4^⁻/⁻^ mice were randomly assigned to two groups (the group sizes are indicated in the corresponding figure legends). In the model group, mice received 5% DSS in drinking water for 5 days followed by normal water. In the treatment group, mice were administered 5% DSS for 5 days followed by normal water, and rhTβ4 (300 μg/mouse/day) was delivered by oral gavage for 7 consecutive days. Body weight and survival were monitored daily.

DSS concentrations were selected according to mouse strain susceptibility and experimental objectives. Higher concentrations (4–5%) were used in C57BL/6 mice, whereas lower concentrations (2–3.5%) were used in BALB/c mice because of their greater sensitivity to DSS-induced colitis. The 3.5% DSS therapeutic model was subsequently used for fibrosis and transcriptomic analyses to maintain experimental consistency.

### Establishment of experimental colitis model and rhTβ4 treatment in WT mice

Male SPF BALB/c mice (8 weeks old) were supplied by Beijing Vital River Laboratory Animal Technology Co. Ltd (Beijing, China). For the prophylactic colitis model, mice were randomly divided into four groups: model, rhTβ4(100), rhTβ4(200), and control (group sizes are indicated in the corresponding figure legends). Beginning on day 0, mice in the rhTβ4 and model groups were treated with 2% DSS in drinking water for 14 consecutive days. During this period, mice in the rhTβ4 (100) group received rhTβ4 (100 μg/mouse/day) by oral gavage, and mice in the rhTβ4 (200) group received rhTβ4 (200 μg/mouse/day) by oral gavage. Mice in the model and control groups received an equal volume of PBS (100 μL) by the same route and schedule. Body weight and survival were monitored daily for 14 days. On day 14, distal colon segments (approximately 2 cm from the anus) were collected for histological examination (H&E staining) and protein extraction for cytokine assays.

In the therapeutic IBD model, the experimental mice were randomly divided into three groups: model, rhTβ4 and control groups (group sizes are indicated in the corresponding figure legends). From day 0, mice in the model and rhTβ4 groups were exposed to 3.5% DSS in their drinking water for 7 days to induce experimental colitis, followed by a recovery phase with normal drinking water. Mice in the control group received standard drinking water throughout the entire experiment. For therapeutic intervention, mice in the rhTβ4 group received rhTβ4 (100 μg/mouse/day, oral gavage) per day from day 8 to day 14. In parallel, mice in the model and control groups were administered 100 μL PBS according to the same route and schedule. Body weight and survival were recorded daily for 14 days. On day 14, mice were euthanized for sample collection. Colonic tissues were harvested, flushed with PBS, and measured for length. Portions of the tissue were allocated for RNA extraction, cytokine analysis, and histological examination.

### Colon histopathological scoring

Colon tissues were fixed in 4% paraformaldehyde, routinely processed, and embedded in paraffin. Serial Sects. (5 μm) were prepared and stained with hematoxylin and eosin (H&E) for histological evaluation.

Histopathological inflammation was assessed using a previously established scoring system [45], which evaluates epithelial damage and inflammatory cell infiltration. Epithelial injury was graded on a scale of 0–4 based on goblet cell depletion and structural integrity of crypt architecture, ranging from normal morphology (0) to extensive crypt loss (4). Inflammatory cell infiltration was similarly scored from 0 to 4 according to the depth and extent of Inflammatory cell infiltration, from no infiltration (0) to transmural involvement extending into the submucosa with marked edema (4).

The total histological score was calculated as the sum of epithelial damage and infiltration scores (total score = E + I).

### Cytokine level assay

Cytokine levels were quantified using the automated Ella system (Bio-Techne) according to the manufacturer’s instructions. Samples were loaded onto the Ella assay plate (SPCKA-MP-003952, Bio-Techne, CA, USA), and cytokine concentrations were measured following the standard protocol.

### Transcriptome sequencing and data analysis

Three biological replicates were included for each experimental group (Control, Model, and rhTβ4), yielding a total of nine RNA libraries. Fresh colon segments were immediately snap-frozen on dry ice and transported to Biomarker Technologies Co. (Beijing, China) for downstream processing.

Total RNA extraction and quality assessment were performed at the company, and only samples passing quality control were used for cDNA library construction. All libraries were sequenced on an Illumina NovaSeq 6000 platform (Illumina Inc., San Diego, CA, USA).

Raw sequencing reads were initially evaluated using FastQC. Adapter sequences and low-quality reads were removed using Cutadapt and Trimmomatic. Clean reads were aligned to the Mus musculus reference genome (GRCm38) using HISAT2 [46]. Gene-level read counts were generated using featureCounts.

Differential expression analysis was performed using the limma package [47]. Raw count data were normalized using the voom transformation prior to linear modeling. *P* values were adjusted for multiple testing using the Benjamini–Hochberg method. Genes with an adjusted *P* value (FDR) < 0.05 and an absolute log_2_ fold change (|log_2_ FC|) > 1 were considered DEGs.

Principal component analysis (PCA) and volcano plots were generated using ggplot2, and heatmaps were generated using the pheatmap package. Gene Ontology (GO) enrichment analysis was performed on intersecting genes using the R package “clusterProfiler” [48].

RNA-seq data have been deposited at Genome Sequence Archive (GSA) under accession number CRA022678.

### RNA extraction and quantitative PCR analysis

Total RNA was isolated from excised colon tissues using TransZol Up (TransGen Biotech, Beijing, China) after tissue homogenization. For cultured cells, total RNA was extracted directly with TransZol Up using the same protocol. RNA concentration and purity were assessed using a NanoDrop spectrophotometer (Thermo Fisher Scientific), and RNA integrity was confirmed by agarose gel electrophoresis.

Complementary DNA (cDNA) was synthesized using PrimeScript reverse transcriptase (TransGen Biotech, Beijing, China) according to the manufacturer’s instructions. Quantitative real-time PCR was performed on an ABI Prism 7300 system using Realtime PCR Supermix with Low ROX (Mei5 Biotechnology Co., Ltd., Beijing, China).

Thermal cycling conditions consisted of an initial denaturation at 95 °C for 30 s, followed by 40 amplification cycles of 95 °C for 5 s and 65 °C for 30 s. Relative mRNA expression levels were calculated using the 2^-ΔΔCt method and normalized to GAPDH. Primer sequences are provided in Supplementary Table S2.

### Masson staining

Deparaffinized colon sections were stained with hematoxylin for 30 s, differentiated in hydrochloric acid until blue, and then sequentially stained with Masson’s staining solution for 2 min, 1% phosphomolybdic acid for 5 min, and bright green staining solution for 5 min. Collagen deposition was observed under a light microscope, and three random fields per section were analyzed using ImageJ to calculate the collagen fiber area.

### Immunohistochemical staining

Formalin-fixed colon tissues were processed using routine paraffin embedding. Serial sections were incubated overnight at 4 °C with primary antibodies against collagen I (1:700) or α-SMA (1:1000). After washing, sections were incubated with a goat anti-rabbit IgG secondary antibody, followed by visualization using diaminobenzidine as the chromogenic substrate. Three random fields per sample were captured, and the expression levels of collagen I and α SMA were quantified using ImageJ software.

### Western blotting analysis

Total protein from colon tissues was extracted after homogenization in RIPA lysis buffer supplemented with PMSF and phosphatase inhibitors (Solarbio, Beijing, China) and incubated on ice for 30 min. Lysates were clarified by centrifugation at 13,500 × g for 15 min at 4 °C, and the resulting supernatants were collected for further analysis.

Protein samples were denatured in 5 × SDS loading buffer and separated by SDS-PAGE, followed by transfer onto PVDF membranes (Merck, Darmstadt, Germany). Membranes were blocked with 5% skim milk for 2 h at room temperature and subsequently incubated with primary antibodies. After washing, membranes were incubated with HRP-conjugated secondary antibodies for 1 h.

Protein bands were detected using SuperKine™ West Femto Maximum Sensitivity Substrate (Abbkine, Wuhan, China) and visualized using a ChemiDoc™ XBS imaging system (Bio-Rad, CA, USA).

### Cell culture and stimulation

Immortalized mouse colon fibroblast cells (MCFCs; NEWGAINBIO, Wuxi, China) were maintained in MEM (Hyclone, UT, USA) supplemented with 10% fetal bovine serum, 100 U/mL penicillin, 100 µg/mL streptomycin, and 2 mM glutamine at 37 °C under a humidified atmosphere with 5% CO₂.

For stimulation experiments, MCFCs were seeded in 6-well plates at a density of 4 × 10^5^ cells per well and allowed to adhere overnight. Cells were then treated with aldosterone (500 nM), rhTβ4 (1 ng/mL), or their combination for 24 h. Cells treated with PBS served as the control group. After treatment, cells were harvested for subsequent qPCR analysis.

### Dual-luciferase reporter assay

MCFCs were seeded in 6-well plates and co-transfected with 0.25 μg of Renilla luciferase control plasmid (pRL-SV40-C; Beyotime Biotechnology, Shanghai, China) and 1 μg of an NR3C2-responsive luciferase reporter plasmid (MR-Luc Reporter; Genomeditech, Shanghai, China) using TurboFect (Invitrogen, Carlsbad, CA, USA), according to the manufacturer’s instructions.

Twelve hours after transfection, cells were treated with aldosterone (500 nM) alone or in combination with rhTβ4 (1 ng/mL) for 24 h. Cells treated with PBS served as the control group.

Luciferase activity was measured using a Dual-Luciferase Reporter Assay System (Promega, Madison, WI, USA) and quantified with a GloMax 96 microplate luminometer (Promega), following the manufacturer’s protocol. Firefly luciferase activity was normalized to Renilla luciferase activity.

### Statistical analysis

Statistical calculations and data visualization were performed using GraphPad Prism 8.0 (GraphPad Software), R (v.4.3.0) and R-studio. Data were expressed as mean ± standard deviation (SD) format with at least 3 replicates in each group. For comparisons between two groups, Student’s t-test was applied. One-way ANOVA analysis was used when comparing 3 or more groups. A *p*-value < 0.05 was considered statistically significant.

## Supplementary Information

Supplementary Material 1.

## Data Availability

The RNA-seq data of this study have been deposited in Genome Sequence Archive (GSA) under accession number CRA022678 and are available at the following URL: https://ngdc.cncb.ac.cn/gsa/browse/CRA022678. Single-cell RNA-seq data were obtained from previously published datasets integrated into the scIBD resource (http://scibd.cn/, accessed 23 May 2025). Publicly available transcriptomic datasets GSE87466 and GSE75214 were obtained from the Gene Expression Omnibus (GEO) database and reanalyzed in this study.
