# Supplementary material for: Recombinant human Thymosin β4 ameliorates experimental colitis and intestinal fibrosis through suppression of mineralocorticoid receptor signaling
Source: Mol Biomed. 2026 Aug 17;7:140. doi: 10.1186/s43556-026-00539-9 (PMC13481925; doi:10.1186/s43556-026-00539-9)
Supplement: Supplementary file 1 [file 43556_2026_539_MOESM1_ESM.docx]

**Title page**

**Recombinant Human Thymosin β4 Ameliorates Experimental Colitis and Intestinal Fibrosis Through Suppression of Mineralocorticoid Receptor Signaling**

Tao-ran Zhao,^1, 2, †^ En-bo Hu,^3, †^ Meng-wei Wang, ^1, 4^ Yan-fang Zhai, ^3^ Yun-yun Mao,^3^ Wen-yi Hou, ^1^ Xiao-zheng Zhang, ^1^ Zhi-zhen Liu, ^1^ Shu-lin Hou, ^1, ***^Jun-jie Xu, ^3, **^ Rui Yu,^3, 5, *^

^1^Department of Biochemistry and Molecular Biology, College of Basic Medical Sciences; Shanxi Key Laboratory of Birth Defect and Cell Regeneration; MOE Key Laboratory of Coal Environmental Pathogenicity and Prevention, Academy of Medical Sciences, Shanxi Medical University, Taiyuan, Shanxi, China.

^2^Shanxi Key Laboratory of Classical Prescription Yang-Strengthening, Taiyuan, Shanxi, China.

^3^ National Key Laboratory of Advanced Biotechnology, Academy of Military Medical Sciences, Beijing, China.

^4^School of Public Health, Shanxi Medical University, Taiyuan, Shanxi, China.

^5^Lead contact

^†^These authors contributed equally

*Correspondence: yurui1102@139.com

**Correspondence: xujunjie@sina.com

***Correspondence: hou_shulin1@163.com

**Supplementary Figure 1**

**Figure S1. Phenotypic characterization of WT and Tβ4⁻/⁻ mice.**

(a) Representative images and quantification of colon length in 8-week-old male WT and Tβ4⁻/⁻ mice (n = 3 per group). (b) Representative H&E staining of colon sections and measurement of crypt length. (c) Immunohistochemical staining for MUC2, Ki67, and BrdU in colon tissues, with quantification of positive cells. (d) Levels of inflammatory cytokines (IL-1β, IL-6, TNF, IFN-γ) measured in colon tissues.

**Supplementary Figure 2**

**Figure S2. In vivo biodistribution of rhTβ4 following oral and intraperitoneal administration**

(a)Representative whole-body fluorescence images of mice at 0, 1, 3, 6, 9, 12, 24, and 48 h after oral administration of Cy5.5-labeled rhTβ4 (rhTβ4-Cy5.5). Images were acquired from the dorsal view and overlaid with photographic images. Control mice received no rhTβ4-Cy5.5. (b)Representative ex vivo fluorescence images of the lungs, stomach, intestine, liver, and kidneys collected at the indicated time points following oral administration of rhTβ4-Cy5.5. Fluorescence images were overlaid with photographic images. (c) Quantification of fluorescence intensity in the intestine following oral administration of rhTβ4-Cy5.5. (d) Representative whole-body fluorescence images of mice at 0, 1, 3, 6, 9, 12, 24, and 48 h after intraperitoneal administration of rhTβ4-Cy5.5. Images were acquired from the dorsal view and overlaid with photographic images. Control mice received no rhTβ4-Cy5.5. (e) Representative ex vivo fluorescence images of the lungs, stomach, intestine, liver, and kidneys collected at the indicated time points following intraperitoneal administration of rhTβ4-Cy5.5. Fluorescence images were overlaid with photographic images. (f) Quantification of fluorescence intensity in the intestine following intraperitoneal administration of rhTβ4-Cy5.5.

**Supplementary Figure 3**

**Figure S3.** **MR(NR3C2) target genes are upregulated in human inflammatory bowel disease datasets.**

(a, b) Heatmaps showing the expression of MR(NR3C2) target genes in two independent human UC datasets: GSE87466 (a) and GSE75214 (b).

**Supplementary Figure 4**

**Figure S4. PCR-based genotyping of Tβ4^-/-^ mice.**

Representative agarose gel electrophoresis results used for genotyping Tmsb4x knockout mice. Lane 1, DNA marker; Lane 2, negative control (water); Lane 3, wild-type (WT) mouse; Lanes 4–11, homozygous Tmsb4x knockout (Tβ4⁻/⁻) mice. Genotypes were determined based on the expected PCR banding patterns. The WT allele generated a single 617-bp band, whereas the knockout allele generated a single 467-bp band. Heterozygous mice exhibited both the 617-bp and 467-bp PCR products. These results confirm successful generation and identification of Tmsb4x-deficient mice.

**Supplementary Table 1**

**Table S1. Summary of scRNA-seq datasets included in this study**

| **Dataset** | **Stage** | **Disease** | **Tissue** | **Sample count** | **Accession** |
| --- | --- | --- | --- | --- | --- |
| K. Parikh et al., Nature, 2019 | Adult | Health, inflamed UC, non-inflamed UC | Colon | 9 | GSE116222 |
| Kinchen et al., Cell, 2018 | Adult | Health, inflamed UC | Colon | 4 | GSE114374 |
| D. Corridoni et al., Nat Med, 2020 | Adult | Health, inflamed UC | Colon | 6 | GSE148837 |
| C. S. Smillie et al., Cell, 2019 | Adult | Health, inflamed UC, non-inflamed UC | Cecum, colon, rectum | 124 | SCP259 |
| J. C. Martin et al., Cell, 2019 | Adult | Inflamed CD, non-inflamed CD | Ileum | 22 | GSE134809 |
| N. Jaeger et al., Nat Commun, 2021 | Adult | Health, inflamed CD, non-inflamed CD | Ileum | 11 | GSE157477 |
| B. S. Boland et al., Sci Immunol, 2020 | Adult | Health, inflamed UC | Ileum, rectum | 21 | GSE125527 |
| M. Friedrich et al., Nat Med, 2021 | Adult | Health, inflamed UC | Colon | 17 | SDY1765 |

Note: SDY1765 is accessible from the Import database (https://www.immport.org/); SCP259 is accessible from Single Cell Portal database (https://singlecell.broadinstitute.org/); The others are accessible from GEO database (https://www.ncbi.nlm.nih.gov/geo).

**Supplementary Table 2**

**Table S1. qPCR primers used in this study**

| Gene | Primers |
| --- | --- |
| *Fkbp5* | Forward Primer: AGGGACTACGAAGCGACAAT  Reverse Primer: GAGAAGCGTTCTGTCCCTGC. |
| *Sgk1* | Forward Primer: ACTGCTCTTCAGTGTGCCCT,  Reverse Primer: GAGGCTGGAAGAAGCAACGG. |
| *Tns2* | Forward Primer: AGATGGGTCTCCCCTCACTC,  Reverse Primer: CAGGGGGAAAGCAGGTGTAG. |
| *Col1a1* | Forward Primer: GAGCGGAGAGTACTGGATCG，  Reverse Primer: GCTTCTTTTCCTTGGGGTTC. |
| *Acta2* | Forward Primer: GTCCCAGACATCAGGGAGTA，  Reverse Primer: TCGGATACTTCAGCGTCAGGA. |
| *GAPDH* | Forward Primer: AGGTCGGTGTGAACGGATTTG,  Reverse Primer: TGTAGACCATGTAGTTGAGGTCA. |

**Materials and Methods for Supplementary Figures**

**Intestinal homeostasis in Tβ4⁻/⁻ mice under steady-state conditions**

To investigate the impact of Tβ4 deficiency on susceptibility to IBD, 8-week-old female and male WT and *Tβ4^⁻/⁻^* mice were administered 4% DSS in drinking water for 5 consecutive days, followed by normal drinking water. Body weight was monitored daily. On day 12, colonic tissues were collected for histological examination (H&E staining) and measurement of inflammatory cytokines. No significant sex-dependent differences were observed; therefore, only male mice were used for subsequent analyses.

**BrdUu incorporation assay for colon epithelial proliferation**

BrdU solution was freshly prepared at 10 mg/mL under sterile conditions. Mice were intraperitoneally injected with 200 μL BrdU per mouse. After 24 h, colons were harvested and fixed in 4% paraformaldehyde, embedded, and sectioned. Immunohistochemical staining was performed to detect BrdU-positive cells, and the number of BrdU-labeled cells in colonic crypts was quantified.

**Immunohistochemical Staining**

Formalin fixed colon tissues were embedded in paraffin routinely. Paraffin sections were incubated with antibodies to Muc2 (1:1000), Ki67 (1:600) overnight at 4 °C. Goat-anti-rabbit IgG was used as a secondary antibody and diaminobenzidine staining was performed. The number of Muc2 or Ki67 positive cells or crypts was determined in 20 random visual fields (200x).

**In vivo biodistribution of rhTβ4 following oral and intraperitoneal administration**

To compare the biodistribution and intestinal retention of recombinant human thymosin β4 (rhTβ4) delivered by different administration routes, eight-week-old male BALB/c mice were obtained from Vital River Laboratory Animal Technology Co., Ltd. (Beijing, China). Mice were randomly assigned to receive Cy5.5-labeled rhTβ4 (rhTβ4-Cy5.5, 100 μL, 1 μg/μL) either by oral gavage or intraperitoneal injection.

Whole-body fluorescence imaging was performed at 0, 1, 3, 6, 9, 12, 24, and 48 h after administration using an IVIS Spectrum in vivo imaging system (PerkinElmer, Waltham, MA, USA) equipped with Cy5.5 filter settings (excitation: 675 nm; emission: 720 nm). At the indicated time points, fluorescence signals were recorded and analyzed to evaluate the distribution and retention of rhTβ4-Cy5.5 in vivo.

To further assess tissue-specific distribution, mice were euthanized at selected time points, and major organs, including the gastrointestinal tract, liver, spleen, lungs, kidneys, and heart, were collected for ex vivo fluorescence imaging using the same imaging parameters. Fluorescence intensity was quantified using Living Image® software (PerkinElmer, Waltham, MA, USA) and expressed as total radiant efficiency within regions of interest (ROIs). The biodistribution profiles obtained from oral gavage and intraperitoneal injection were compared to determine the optimal route for subsequent colitis studies.
